# Using patients’ oral photographs for oral hygiene motivation promotes periodontal health: a prospective randomized controlled trial

**DOI:** 10.1186/s12903-024-04553-7

**Published:** 2024-07-13

**Authors:** Didem Özkal Eminoğlu, Beyza Nur Şahin, Dilek Biçer, Gülsüm Dal, Ismayıl Huseynlı, Anvar Dadashov, Didar Betül Doğan, Kamber Kaşali

**Affiliations:** 1 Faculty of Dentistry, Department of Periodontology, Atatürk University, Erzurum, Turkey; 2 Faculty of Medicine, Department of Biostatistics, Atatürk University, Erzurum, Turkey

**Keywords:** Oral hygiene, Periodontal health, Periodontal indices, Motivation, Modified Bass technique

## Abstract

**Objectives:**

The aim of this prospective, randomized, controlled, single-centered, examiner-blinded clinical trial was to evaluate the effectiveness of a personalized and visual oral health education program in addition to conventional oral hygiene education.

**Materials and methods:**

Fifty-six non-smoker, right-handed participants (aged 30.34 ± 11.46 years) without clinical signs of periodontitis were randomly grouped: the intervention group (*n* = 28) received a personalized visualized oral health education combined with conventional oral hygiene education, and the control group (*n* = 28) received conventional oral hygiene education only. All participants were assessed for improved periodontal parameters (PI, GI, BOP, and PPD) at baseline, first month, and third month.

**Results:**

A significant reduction (*p* < 0.001) was observed in PI, GI, and BOP during two follow-up sessions compared to the baseline for the two groups. No differences were found for inter-group (*p* > 0.05) or intra-group (*p* = 1) comparison of PPD. PI (*p* = 0.012), GI (*p* = 0.032), and BOP (*p* = 0.024) scores were significantly reduced at the third-month follow-up assessment in the intervention group compared to the control group.

**Conclusions:**

Clinical outcomes of periodontal health were significantly enhanced by the personalized and visual oral health education program applied in this study compared to the conventional oral hygiene education program.

**Clinical relevance:**

Numerous studies reported additional interventions to the oral hygiene education program. However, we did not find any published studies investigating the role of patients’ intra-oral photographs in oral care. This study’s results demonstrated that a visually aided education program for oral hygiene motivation may help improve oral health.

**Clinical trial registration:**

Registration number is “NCT06316505” and date of registration is 18/03/2024.

## Introduction

Oral health is the health state of the oral cavity and related tissues that enables a person to eat, speak, and communicate effectively without any active disease or discomfort; it is linked to general health and life quality [1]. Poor oral health negatively affects life quality by causing pain and discomfort, malnutrition, and absenteeism at school/work, and can lead to infectious and degenerative diseases. Oral health is crucial for individuals and society as a whole. However, knowledge of the effective promotion of oral care is scarce [2]. The World Health Organization (WHO) is actively engaged in efforts to promote global awareness regarding oral health [3].

Dental caries and periodontal disease are the most common diseases worldwide and are preventable. Tooth loss from these conditions is a common clinical indicator of poor oral health and affects a large proportion of the world population [4]. Periodontal diseases are chronic inflammatory and infectious disorders that can occur in individuals of all age groups, with varying prevalence rates among various populations. Gingivitis and periodontitis affect 48.5–88% and 5–15% of the world population, respectively [5, 6]. The prevalence rates in Turkey are 49–72% for gingivitis and 2–46.4% for periodontitis [7, 8].

Studies have demonstrated that dental plaque is the major cause of periodontal diseases. This progressive inflammatory condition is caused by bacteria, toxins, enzymes, and metabolites in microbial dental plaque [9, 10]. To prevent periodontitis, controlling gingivitis by maintaining proper oral hygiene is essential. Implementing personal daily oral hygiene routines is the most critical determinant in preventing and managing periodontal disease [11, 12]. While the oral cavity’s natural cleansing mechanisms may eliminate food particles, they cannot efficiently eliminate the supragingival plaque, which requires consistent oral hygiene practice. Tooth brushing is the most widely used mechanical method for plaque control. Additionally, interdental cleaning procedures and chemical plaque control are major methods for plaque removal [13]. Effective dental hygiene can be maintained by utilizing a suitable toothbrush twice a day to control plaque accumulation.

Maintaining oral health requires adherence to a lifelong oral hygiene regimen, which is significantly influenced by the individual’s motivation and manual dexterity. The dentist’s responsibility in this context is to provide guidance to the patient regarding the appropriate and efficient oral hygiene routine and to motivate their adherence to it [14]. Motivation aims to stimulate action or effort toward a particular goal or objective. It is a condition that can be altered in response to external influences and time rather than a characteristic. The primary objective of motivation is to enhance people’s knowledge by highlighting oral health, introducing oral hygiene, and offering information on efficient mechanical cleaning procedures to eliminate risk factors for poor oral hygiene that contribute to its deterioration [15]. Individuals’s motivation is at its highest level in the first days of training and decreases over time; therefore, an appropriate interval of appointment specific to the individual should be planned. Generally, three months is the most preferred appointment time frame [16].

Frequently employed motivation strategies include chair-side instructing, message reminders, modifying behavior, and the Hawthorne effect. Verbal and written instructions, photographs or catalogs, videos, and visual demonstrations with models or experimental equipment were employed in the chair-side approach to increase oral hygiene motivation among patients [17]. The techniques for promoting oral hygiene also involve text messages [18, 19], integrating artificial intelligence in mobile health applications [20], and launching a WhatsApp-based chat room for participants to post tooth selfies [21].

Medical research suggests that utilizing visual aids in healthcare can enhance patients’ comprehension and motivation, resulting in more enduring changes in mindsets and behavioral intentions [22, 23]. Although it demands a significant amount of chair time, visual aids can save time in the long run. Better-motivated patients spend less time in follow-up sessions and display better treatment results [12].

Based on these considerations, this randomized controlled trial aimed to determine the effectiveness of a personalized and visual oral health education program in addition to conventional education on periodontal parameters. The null hypothesis of the study is that using oral photographs of patients in addition to conventional oral health education has no effect on oral hygiene.

## Materials and methods

### Study design, registration, ethical approvals

This clinical trial was designed as a randomized, controlled, examiner-blinded study with two parallel arms. Before the study, each participant signed an informed consent form. The study was conducted in accordance with the guidelines of the World Medical Association Declaration of Helsinki of 1975, as revised in 2013. The ethical approval was obtained from “The Ethics Committee of the Faculty of Dentistry at Ataturk University” (meeting date: 23.11.2023; meeting number: 11; decision no: 58). The study design was based on the CONSORT (Consolidated Standards of Reporting Trials) 2010 statement. The clinical trial registration number is “NCT06316505,” and the date of registration is 18.03.2024.

The study was conducted at the Atatürk University Faculty of Dentistry, Department of Periodontology Clinic, Erzurum, Türkiye, between December 2023 and March 2024. It included a baseline data collection and two follow-up assessments.

### Study population and intervention

The subjects were recruited among patients attending periodontal health care. The inclusion criteria were age between 18 and 60 years, 12 teeth minimum, and availability for follow-up assessments. The study excluded participants with systemic diseases or conditions, neurological or psychiatric disorders, physical or mental disabilities, smoking habits, or medication use [24, 25] that could potentially impact their periodontal health. Individuals with a history of periodontal disease treatment or recent usage of antibacterial mouth rinses in the preceding six months were also excluded. For standardization, only right-handed patients at toothbrushing were selected.

Participants were divided into two groups:

Control group (CG) (*n* = 28): conventional oral hygiene education (COHE) [26].

Intervention group (IG) (*n* = 28): in addition to COHE, the following steps were followed:

- Session 1 [baseline (T0)]: a photograph was taken before (F1) and after (F2) full-mouth scaling root planing (fmSRP), and the patient was presented with the difference.
- Session 2 [one month after baseline) T1]: a photograph was taken (F3), and the patient was presented with the difference between F1 and F3.
- Session 2 [(three months after baseline) T2]: a photograph was taken (F4), and the patient was presented with the difference between F1, F3, and F4.

The COHE was provided by DBD for each participant one by one and face to face. The tooth cleaning techniques (modified Bass technique and interdental brushing) [27] were also demonstrated in each session (T0, T1, and T2). The patients were requested to brush their teeth in front of a mirror after the demonstration of brushing procedures. Each participant was provided with an identical toothbrush and toothpaste to minimize potential effects on oral hygiene. All participants were recommended to brush twice daily for two minutes [28] and encouraged to adhere to oral self-care recommendations to attain optimal oral health [29]. The procedure lasted approximately 20 min and was performed in a quiet environment [30].

Dental photographs [31] were taken (by AD) at each session (T0, T1, and T2). Each participant’s intraoral images were photographed using a digital camera equipped with a ring flash and a 100-mm macro lens. Five photos were taken of each participant. Images of the frontal side display the labial surfaces of the front teeth. Lateral images taken from the right and left sides display the buccal surfaces of the posterior teeth. Photographs of the maxillary and mandibular dentition present the palatal/lingual and occlusal surfaces [32].

The intervention group received visual motivation in addition to COHE by being presented with photographs (F1, F2, F3, and F4) of the changes in their oral health status taken at each session.

### Oral assessment

Parameters were measured blindly by researchers. Oral assessments were recorded by four dentists [33] (IH, BNŞ, DB, and GD), who were trained and calibrated by an experienced calibrated examiner (DÖE) for periodontal measurements. For intra-investigator calibration, the investigators repeated periodontal measurements at baseline and one week later for a number of patients equal 10% of the participants. These patients were not included in the study. Intra-rater correlations were PI: ICC = 1.000, *p* < 0.001; BOP: ICC = 1.000, *p* < 0.001; PPD: ICC = 0.828, *p* = 0.038; GI: ICC = 0.995, *p* < 0.001. The intervention and control groups were assessed at baseline (T0), first follow-up (T1) [34], and second follow-up (T2) [35]. The plaque index (PI) [36], gingival index (GI) [37], bleeding on probing (BOP) [38], and periodontal probing depth (PPD) were measured at T0, T1, and T2 using the Williams periodontal probe (Hu-Friedy, Chicago, IL, USA).

### Sample size

G-Power^®^ version 3.1 (University of Düsseldorf, Düsseldorf, Germany) was used for sample size estimation. The study’s power analysis was based on a previous study [39]. The patient’s oral hygiene status was calculated based on the Silness-Löe Plaque Index [36] and on the change in plaque index scores at the baseline (mean ± standard deviation = 1.14 ± 0.51) and third month (expected mean ± standard deviation = 0.85 ± 0.51) in the motivation group. Accordingly, the expected mean difference and effect size were 0.29 and 0.560, respectively. Twenty-eight volunteers were included in each group for a type I error of 5% and a power of 80%.

### Randomization and blinding

Participants were divided into two groups using the https://www.randomizer.org/ website. A research randomizer is a tool that generates random numbers to allocate people for clinical trials. This software can be classified as a “pseudo-random number generator,” like many other computer-based generators. It utilizes a sophisticated algorithm initialized by the computer’s clock to produce random numbers [40]. The oral hygiene instructor, clinical examiners, and statistical analyzer were all blinded to the groups of the patients.

### Statistical analysis

Analyses were performed (by KK) with the IBM SPSS 20 statistical analysis program. Data are presented as mean ± standard deviation, median, minimum, maximum, percentage, and number. The normal distribution of continuous variables was analyzed using the Shapiro–Wilk test, Kolmogorov–Smirnov test, Q-Q plot, skewness, and kurtosis. If normal distribution conditions were met when comparing two independent groups, the independent samples *t*-test was used; otherwise, the Mann–Whitney *U* test was employed. When comparing more than two dependent group variables, the Repeated Measures ANOVA test was used if the distribution was normal; otherwise, the Friedman test was used. Sphericity Assumed or Greenhouse-Geisser methods were used according to the presence of sphericity in the Repeated Measures test. Post-hoc tests were performed after the Repeated Measures test using Tukey’s test when variances were homogeneous and Tamhane’s T2 test when they were not. For post-hoc tests after the Friedman test, a Friedman 2-way ANOVA by ranks (*k* samples) test was used. For 2 × 2 comparisons between categorical variables, the Pearson Chi-square test was used if the expected value was > 5, chi-square Yates test if the expected value was between 3 and 5, and Fisher’s Exact test if the expected value was < 3. For comparisons larger than 2 × 2 between categorical variables, the Pearson Chi-square test was used when the expected value was > 5, and the Fisher-Freeman-Halton test was used when the expected value was < 5. In the comparison of two quantitative variables, the Pearson correlation test was used if the normal distribution condition was met; otherwise, the Spearman correlation test was used. The statistical significance level was set at *p* < 0.05.

## Results

**Fig. 1:**
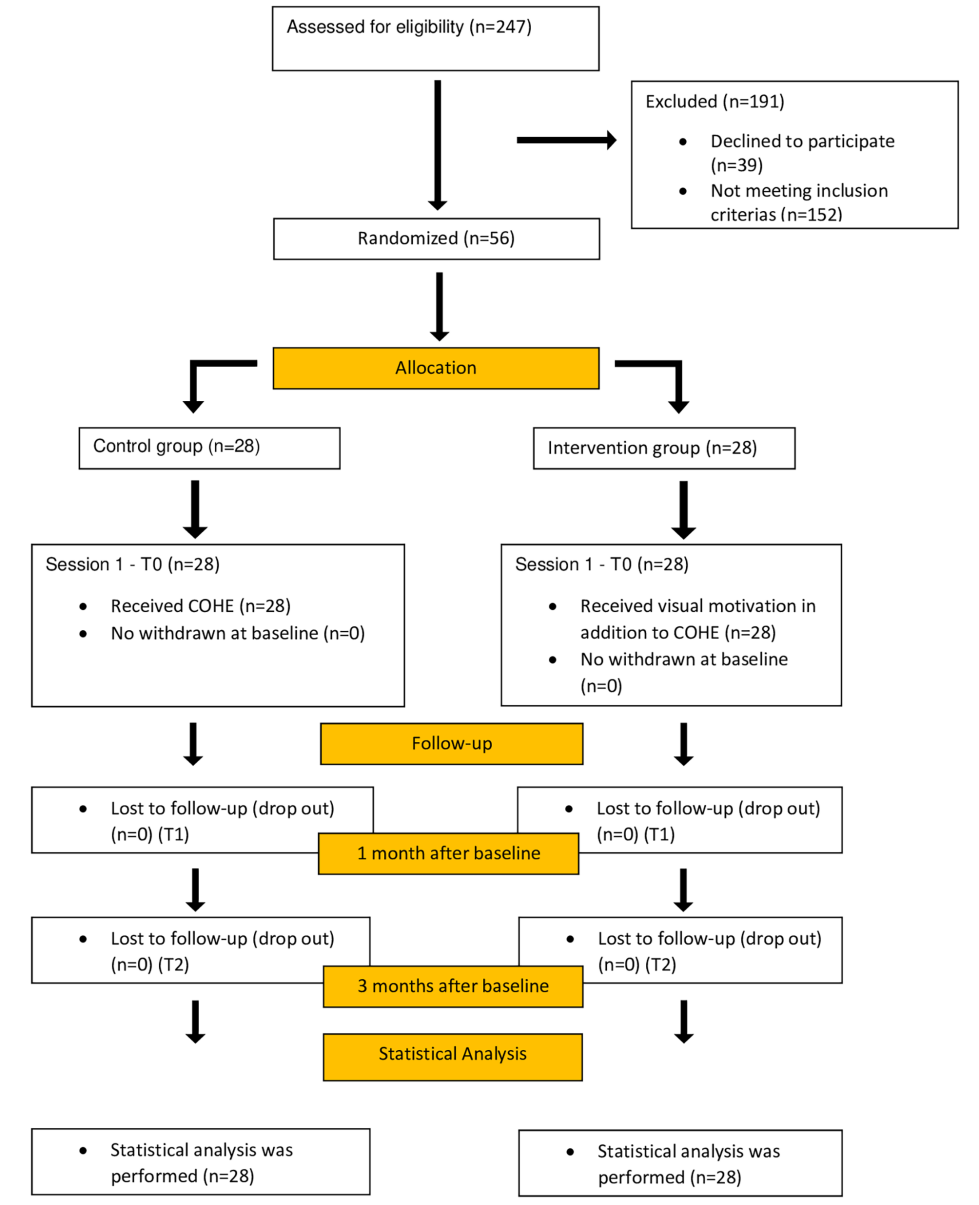
CONSORT flow chart

247 patients were assessed for eligibility. Among them, 191 participants were excluded from the study (39 declined to participate, and 152 did not meet the inclusion criteria). 58 participants were enrolled in the study and randomly separated into two groups (*n* = 28). Over the follow-up period (first and third months), no one dropped out (Fig. 1).

A comparison of demographic characteristics at the baseline revealed no significant differences [age (*p* = 0.325), gender (*p* = 0.057), and educational level (*p* = 0.756)] between the two groups (Table 1).

**Table 1:** Demographic characteristics of participants

Demographic characteristics		Groups				*p*
		IG (*n* = 28)		CG (*n* = 28)		
		n	%	n	%	
**Gender**	Female	15	53.6	8	28.6	0.057 ^£^
	Male	13	46.4	20	71,4	
**Educational level**	Primary School	6	21.4	6	21.4	0.756 ^£^
	High School	8	28.6	6	21.4	
	University Degree	13	46.4	15	53.6	
	Postgraduate	1	3.6	1	3.6	
		**Mean ± sd; median**		**Mean ± sd; median**		***p***
**Age**		32.04 ± 12.29; 30		28.64 ± 10.51; 26		0.325 ^β^

*p* value considered significant when *p* < 0.05; ^β^: Mann-Whitney U test; ^£^: Chi-square test; IG: intervention group; CG: control group

No correlation was found between age and oral hygiene parameters measured in all sessions (*p* > 0.05) (Table 2). No significant difference was observed between gender and oral hygiene parameters measured in all sessions (*p* > 0.05).

**Table 2:** Correlations between age and clinical periodontal parameters

			PI (T0)	PI (T1)	PI (T2)	GI (T0)	GI (T1)	GI (T3)	BOP (T0)	BOP (T1)	BOP (T2)	PPD (T0)	PPD (T1)	PPD (T2)
**Spearman’s rho**	**Age**	**r**	-0.016	-0.039	0.010	-0.043	-0.004	0.149	0.104	0.140	0.140	0.153	0.010	-0.043
		**p**	0.908	0.774	0.942	0.764	0.976	0.272	0.452	0.302	0.302	0.282	0.942	0.764
		**N**	56	56	56	56	56	56	56	56	56	56	56	56

*p* value considered significant when *p* < 0.05 (2-tailed). r: Spearman correlation coefficient

T0: baseline, T1: 1 month after baseline, T2: 3 months after baseline

PI: plaque index, GI: gingival index, BOP: bleeding on probing, PPD: periodontal probing depth

PI (*p* = 0.491), GI (*p* = 0.557), BOP (*p* = 0.466), and PPD (*p* = 0.529) revealed no significant differences at T0 (Table 3). A statistically significant difference was observed between T0 and T1 and between T0 and T2 outcomes in the two groups for PI, GI, and BOP (*p* < 0.001). No statistically significant difference was observed for PPD scores between T0, T1, and T2 outcomes in the two groups (*p* = 1.0). Periodontal variables revealed no significant differences [PI (*p* = 0.491), GI (*p* = 0.557), BOP (*p* = 0.466), and PPD (*p* = 0.529)] between the two groups at T0. After three months, the intervention group displayed significant improvements in PI (*p* = 0.012), GI (*p* = 0.032), and BOP (*p* = 0.024) than the control group. In contrast to the changes seen in other periodontal parameters, no statistically significant difference (*p* = 0.503) was observed between the two groups in the mean scores of PPD measured three months after T0.

**Table 3:** Clinical periodontal parameters at the baseline, 1st month, and 3rd month

		Groups				*p* ^β^
		IG (*n* = 28)		CG (*n* = 28)		
Clinical indices	Time frame	Mean ± Standard Deviation	Median (minimum – maximum)	Mean ± Standard Deviation	Median (minimum – maximum)	
**PI**	T0	1.76 ± 0.59 ^a^	1.57 (0.78-3.0)	1.79 ± 0.65 ^a^	1.88 (0.17–2.64)	0.491 ^β^
	T1	0.87 ± 0.59 ^b^	1.0 (0–3.0)	1.08 ± 0.54 ^b^	1.04 (0.14-2.0)	0.079 ^β^
	T2	0.57 ± 0.51 ^b,£^	0.42 (0–2.0)	0.92 ± 0.51 ^b,¥^	1.0 (0.07-2.0)	0.012 ^β^
	*p* ^≠^	< 0.001 ^≠^		< 0.001 ^≠^		
**GI**	T0	1.33 ± 0.63 ^a^	1.33 (0–2.0)	1.29 ± 0.55 ^a^	1.33 (0–2.0)	0.557 ^β^
	T1	0.8 ± 0.5 ^b^	1.0 (0-1.83)	0.86 ± 0.58 ^b^	1.0 (0–2.0)	0.520 ^β^
	T2	0.43 ± 0.4 ^b,£^	0.32 (0-1.07)	0.74 ± 0.51 ^b,¥^	1.0 (0-1.5)	0.032 ^β^
	*p* ^≠^	< 0.001 ^≠^		< 0.001 ^≠^		
**BOP**	T0	54.57 ± 38.2% ^a^	47.9 (0-100) %	45.36 ± 31.9% ^a^	38.22 (0-100) %	0.466 ^β^
	T1	18.41 ± 23.95% ^b^	11.5 (0-100) %	21.77 ± 25.84% ^b^	14.54 (0-100) %	0.426 ^β^
	T2	4.36 ± 10.24% ^b,£^	0 (0–50) %	11.96 ± 15.41% ^b,¥^	4 (0–57) %	0.024 ^β^
	*p* ^≠^	< 0.001 ^≠^		< 0.001 ^≠^		
**PPD**	T0	2.13 ± 0.49	2.0 (2.0-4.07)	2 ± 0.01	2.0 (2.0-2.03)	0.529 ^β^
	T1	2.13 ± 0.49	2.0 (2.0-4.07)	2 ± 0.01	2.0 (2.0-2.03)	0.529 ^β^
	T2	2.15 ± 0.52	2.0 (2.0-4.07)	2 ± 0.01	2.0 (2.0-2.03)	0.503 ^β^
	*p* ^≠^	1.000 ^≠^		1.000 ^≠^		

^β^: Mann Whitney U test was used for intra-goup comparisions. ^a, b^ means intra-group statistical significance by post-hoc test results

^≠^: Friedman test was used for inter-goup comparisions.^£,¥^means inter-group statistical significance

*p* value considered significant when *p* < 0.05, IG: intervention group, CG: control group

T0: baseline, T1: 1 month after baseline, T2: 3 months after baseline

PI: plaque index, GI: gingival index, BOP: bleeding on probing, PPD: periodontal probing depth

## Discussion

This study aimed to examine the impact of a personalized visual oral hygiene motivation program in addition to regular oral hygiene instruction on clinical periodontal parameters.

Studies have demonstrated that adopting effective brushing techniques is more crucial than choosing the right brushing instrument to manage dental plaque. Throughout the last half-century, other brushing techniques have emerged, but only the Bass technique has proven effective in cleansing the gingival sulcus. The modified Bass technique is frequently suggested despite being challenging to learn and execute. However, no universally agreed-upon brushing approach is currently available for optimal plaque removal [41]. Therefore, the present study employed the modified Bass technique in its oral hygiene education program.

Participants were recommended to brush their teeth twice daily for two minutes [28]. Findings indicate that those following this recommendation and establishing a habit from their early years experience a lower incidence of tooth decay. Based on the results of the review conducted by Slot et al., an average of 42% of dental plaque can be eliminated with two minutes of manual brushing. Research has demonstrated that a brushing time of two minutes yields superior results compared to one minute [42]. Extending the brushing time to three minutes or more does not seem to enhance the efficacy of plaque removal and is likely to discourage most participants [43].

The hand preference of the individuals participating in this study for brushing was taken into consideration. The literature indicates that right-handers clean their left jaw better than their right jaw, while left-handers are more successful in the right jaw than the left one [44]. All selected participants were right-handed for standardization.

Several strategies have been developed to aid oral hygiene education [20]. Nevertheless, despite consistent and frequent professional reminders given directly to patients during dental appointments, modifying their oral hygiene behaviors tends to be challenging. AI can improve personnel behaviors when utilized with digital technologies, like augmented and virtual reality or mobile health apps. Yet, chair-side instruction may still be the gold standard for oral hygiene education [45, 46]. This study’s results indicate that oral health education based on a conventional method, with or without visual materials, effectively improves oral hygiene. PI, GI, and BOP were significantly improved from T0. The improved oral hygiene may be due to periodic training. Additionally, the Hawthorne effect should be considered [47].

The ameliorative effect of the educational program on oral health behaviors was verified by decreasing PI scores over the study period in the two groups. The plaque indices significantly improved during the first month of the study. The initial reduction in the dental plaque amount in the two groups can be explained by the fmSRP itself. Professional plaque removal by dentists may affect the first-month PI scores. Additionally, a significant improvement in PI was observed in the two groups after three months compared to the baseline. The existing literature strongly supports the notion that participants in clinical trials exhibit an enhanced ability to develop certain habits when follow-up sessions are implemented [47]. The similar plaque reduction in the intervention and control groups during the study period is consistent with reports indicating a reduced PI after an oral hygiene education program [33, 48]. The two groups also displayed a significant decrease in BOP and GI at the two follow-up sessions compared to the baseline, which may be related to reduced plaque accumulation. The BOP presence is a major clinical periodontal parameter and the most important sign of gingivitis. Therefore, its reduction is a sensitive and reliable indicator of a positive change in oral care [49]. A systematic literature review reported that an oral hygiene motivation program in patients undergoing fixed orthodontic treatment leads to reduced microbial dental plaque accumulation and improved gingival health [50]. The present results also confirm that personalized education has a more positive effect on PI, GI, and BOP after three months than standardized oral hygiene instructions. These results are similar to studies comparing conventional and personalized oral hygiene education that proved the advantage of individualized instruction methods [51, 52]. The present results are in line with Rohsenow et al.’s [53] recommendation to employ a direct counseling method to motivate patients. This means that oral hygiene education should pay attention to the individual strengths and weaknesses of patients and implement a motivational program accordingly. A qualitative study demonstrated that creating a personalized dialogue and a good relationship between the caregiver and the patient affects lifestyle habits positively [54].

This study demonstrated that scaling, root planing, and oral hygiene instructions do not affect the stability of shallow PPD. This could be related to the fact that the mean PPD scores recorded at the initial session for the groups were under 3 mm. The study population included individuals with no clinical signs of periodontitis. The initial absence of a pathologic probing depth at the beginning of the trial may be the reason for the absence of a reduction in pocket depth.

### Study limitations and perspectives

The study was conducted on a relatively small sample size, and the participants were only observed for a short time frame of three months; therefore, the findings may not provide information about potential long-term outcomes. Different operators involved in the oral assesment is another limitation of the study. The Hawthorne effect is another limitation of studies involving participants’ motivation. Future studies should include a larger sample size involving patients with periodontitis and an extended observation period with other brushing techniques and different toothbrushes. Future studies could also involve elderly participants since age is a crucial factor involving cognitive abilities [30].

## Conclusions

The two groups displayed a significant reduction in PI, GI, and BOP scores. The beneficial effect of the client-centered, directive therapeutic, visually aided oral health education approach significantly improved clinical outcomes compared to conventional oral hygiene education alone.

## Data Availability

Data is provided within the manuscript or supplementary information files.
